# Direct ChIP-Seq significance analysis improves target prediction

**DOI:** 10.1186/1471-2164-16-S5-S4

**Published:** 2015-05-26

**Authors:** Mukesh Bansal, Geetu Mendiratta, Santosh Anand, Ritu Kushwaha, Ryan Hyunjae Kim, Manju Kustagi, Archana Iyer, Raju SK Chaganti, Andrea Califano, Pavel Sumazin

**Affiliations:** 1 Department of Systems Biology, and Center for Computational Biology and Bioinformatics, Columbia University, New York, NY, USA; 2 Cancer Genetics Inc, New Jersey, USA; 3 Department of Science and Technology, University of Sannio, Benevento, and Institute for Biomedical Technologies, National Research Council, Milan, Italy; 4 Memorial Sloan-Kettering Cancer Center, 1275 York Avenue, New York, NY, USA; 5 Laboratory of Immune Cell Epigenetics and Signaling, Rockefeller University, 1230 York Avenue, New York, NY 10065., USA; 6 Institute for Cancer Genetics, Columbia Genome Center, High Throughput Screening facility, Department of Biomedical Informatics, Department of Biochemistry and Molecular Biophysics, and Herbert Irving Comprehensive Cancer Center, Columbia University, New York, NY, USA; 7 Texas Children's Cancer Center, Baylor College of Medicine, Houston, Texas 77030, USA

**Keywords:** ChIP-Seq, peak calling, protein-DNA binding sites

## Abstract

**Background:**

Chromatin immunoprecipitation followed by sequencing of protein-bound DNA fragments (ChIP-Seq) is an effective high-throughput methodology for the identification of context specific DNA fragments that are bound by specific proteins *in vivo*. Despite significant progress in the bioinformatics analysis of this genome-scale data, a number of challenges remain as technology-dependent biases, including variable target accessibility and mappability, sequence-dependent variability, and non-specific binding affinity must be accounted for.

**Results and discussion:**

We introduce a nonparametric method for scoring consensus regions of aligned immunoprecipitated DNA fragments when appropriate control experiments are available. Our method uses local models for null binding; these are necessary because binding prediction scores based on global models alone fail to properly account for specialized features of genomic regions and chance pull downs of specific DNA fragments, thus disproportionally rewarding some genomic regions and decreasing prediction accuracy. We make no assumptions about the structure or amplitude of bound peaks, yet we show that our method outperforms leading methods developed using either global or local null hypothesis models for random binding. We test prediction performance by comparing analyses of ChIP-seq, ChIP-chip, motif-based binding-site prediction, and shRNA assays, showing high reproducibility, binding-site enrichment in predicted target regions, and functional regulation of predicted targets.

**Conclusions:**

Given appropriate controls, a direct nonparametric method for identifying transcription-factor targets from ChIP-Seq assays may lead to both higher sensitivity and higher specificity, and should be preferred or used in conjunction with methods that use parametric models for null binding.

## Background

Chromatin Immunoprecipitation (ChIP) is widely used to discover *in-vivo *protein-bound or epigenetically modified DNA regions, including binding sites for transcription factors [1,2] and chromatin modification enzymes [3]. Knowledge about these sites and interactions lays the foundation for investigating cellular processes in a variety of contexts, including processes that guide development and respond to stimuli. Protocols for ChIP vary, but in general, after protein cross-linking and DNA fragmentation, specific antibodies are used to selectively immunopurify protein-bound DNA fragments, whose identity and abundance are then determined by quantitative PCR (ChIP) [4,5], microarray hybridization [6] (ChIP-chip) or genome-wide sequencing [1] (ChIP-Seq).

ChIP-Seq allows identification of bound DNA sites anywhere in the genome and has thus become the method of choice for genome-wide profiling of protein binding sites. ChIP-Seq is routinely used to screen for transcription factor (TF) targets and binding sites, identifying TF targets that may be dysregulated by changes to TF expression and activity. Despite its popularity, however, this method continues to pose unique data-analysis challenges, due to the significantly larger amount of generated data and technological bias. The assays used by this approach generally produce millions of DNA fragment reads that were potentially bound to a protein of interest, as well as large amounts of background fragment reads that can contribute to false-positive target predictions. Consequently, analysis methods find a balance between false-positive and false-negative error rates, where false-positive predictions need to be weeded out by additional assays and false-negatives may result in missed interactions that are informative about regulation in the screened cells.

Typically, reads are first aligned to a reference genome, producing consensus regions with multiple matching fragment reads. These are used to produce genome occupancy histograms, whose peaks are then analyzed to evaluate their statistical significance, as the likelihood that they were produced by random chance events (null hypothesis), based on relatively simple models for random fragment overlap, such as Poisson processes. Finally, the *peak-calling *process separates those DNA regions that are likely to be bound by the specific protein, at a given statistical significance threshold, from those that are more likely to correspond to false positive events. A problem of most such approaches for null hypothesis evaluation is that they use global probabilistic models that may fail to fully account for both technical and biologically-motivated local fluctuations in binding probability.

Peak-calling methods that compute false positive likelihood (null hypothesis) by modeling chance DNA fragment immunoprecipitation using a Poisson process include MACS [7], USeq [8], QuEST [9], and SISSRs [10]. Alternative null hypothesis evaluation methods have been proposed, including the use of a negative binomial distribution [11], binomial distributions that are conditional on data from control experiments [8,12], and hidden Markov models [13]. All of these approaches adopt models that are uniformly computed across the genome, subsequently failing to identify many relevant binding regions [14,15]. PeakFinder [16], for instance, calculates peak significance using a genome-wide Poisson distribution, thus discounting local bias.

To address some of these issues, MACS [7]combines genome-wide and local models, by comparing candidate peaks to an *a priori *null-model background derived from genome-wide and local flanking regions, as well as from control experiments. In contrast to the approach taken by MACS, we take advantage of control experiments, when available, to generate a fully unbiased, non-parametric model, where no peak magnitude distribution is assumed *a priori*. We then analyze whether such a model may be effective in identifying *bona fide *binding sites that are missed by PeakFinder and MACS, while maintaining high specificity.

**Figure 1:**
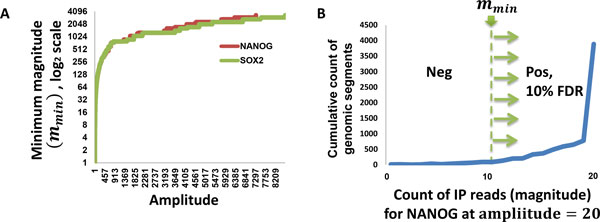
**dIP significance estimates for bound genomic regions depend on both experiments (IP) and control**. Significance is evaluated using the number of IP fragments (magnitude) after conditioning for the total number of fragments aligned to the region (amplitude) in both IP and control. (A) Minimum magnitudes for NANOG and SOX2 IP as a function of the amplitude to obtain FDR ≤ 0.1. (B) Minimum magnitude (*m_min_*) for a fixed amplitude is the magnitude necessary for achieving statistical significance at a given FDR cutoff. It is calculated as that value of m at which the % of cumulative regions just crosses the FDR value. Here we present IP read count for regions with amplitude 20 in NANOG ChIP-Seq data.

The dIP algorithm (differential ImmunoPrecipitation) evaluates candidate DNA fragment consensus peaks using a nonparametric genome-wide approach, after conditioning on non-specific binding in control experiments. dIP proceeds by first building a null distribution for non-specific immunoprecipitation based on control experiments to evaluate the statistical significance of candidate consensus peaks (magnitude in Figure 1A) based on the total number of DNA fragments supporting their occupancy across both specific and non-specific (control) antibody assays (amplitude in Figure 1A). After partitioning the genome into overlapping same-length segments, dIP first evaluates each segment relative to others with similar representation in the IP and control experiments, choosing a minimum magnitude for each amplitude (Figure 1B); note that the minimum magnitude is monotonically coupled with the desired FDR cutoff, i.e. choosing a higher minimum magnitude improves specificity at the expense of sensitivity, see Methods for details. dIP then joins neighboring segments with evidence for binding by the immunoprecipitated protein to produce contiguous target region predictions. We show that this intuitive scoring approach identifies sites that are missed by PeakFinder and MACS, without increasing false positive calls, thus comparing favorably with these algorithms both in terms of predictive power and cross-platform reproducibility. Our results suggest that unbiased comparison against non-specific controls can help to significantly improve the accuracy of ChIP-Seq analysis.

## Results and discussion

To evaluate dIP's performance, we compared the binding sites inferred by dIP, PeakFinder, and MACS using multiple ChIP-Seq datasets. The performance assessment was done using multiple metrics, including enrichment of functional targets using silencing datasets, concordance of predictions obtained from other technologies and reproducibility/consistency in predictions using replicated datasets. For evaluation we used ChIP-seq data for NOTCH1 in TALL cells [2,17], and NANOG and SOX2 in the germ-cell tumor cell line NT2/D1[18], ETS1 in Jukart cells [19]and FOXA1 in MCF7 cells [20]. To facilitate cross-platform comparison and functional validation, we restricted our analysis of gene-target predictions to sites within 2 Kb from canonical transcription start sites.

### Predictions

We compared the number of predicted target genes for NOTCH1, NANOG and SOX2 ChIP-Seq data according to dIP, MACS and PeakFinder. For this comparison, we identified all the genes with predicted target regions within 2 kb of the TSS. While dIP and MACS identified substantial number of target genes (~2000) for both NANOG and SOX2, PeakFinder identified very few target genes (less than 100). Also we found significant variability in number of identified targets by PeakFinder, where the number of identified targets varied from under 20 to greater than 6000 for NOTCH1. Consequently, we removed PeakFinder from further comparisons.

**Figure 2:**
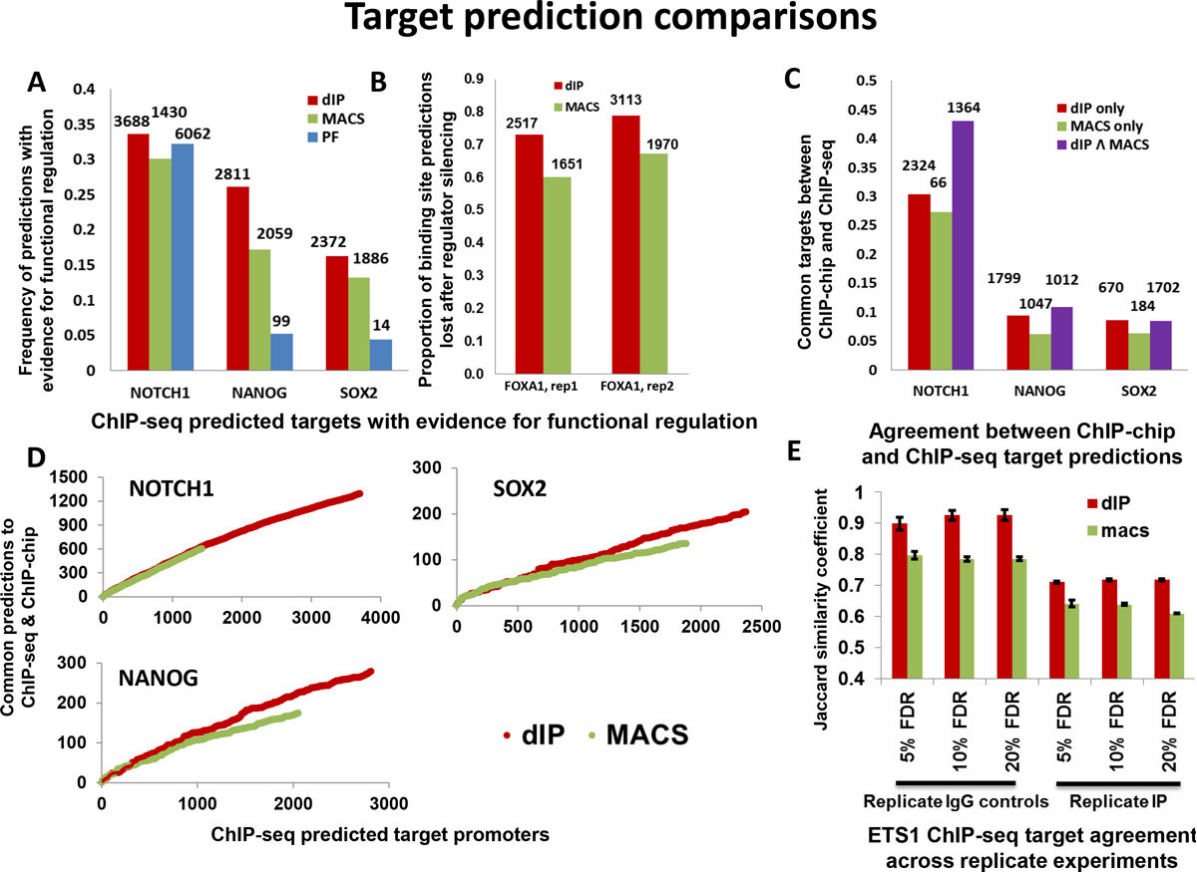
**Concordance between promoter occupancy predictions, and evidence for functional regulation and ChIP-chip predictions**. (A) The count of predicted promoters bound by NOTCH1, NANOG and SOX2 from ChIP-Seq data is given as data labels, while the proportion of associated genes with evidence for functional regulation from RNAi studies is given on the y-axis. (B) The number of predicted targets for FOXA1 is given as data labels, y-axis reports on the proportion of target predictions lost after TF silencing. (C) Common target gene predictions from ChIP-chip and ChIP-Seq, where data labels give the absolute count of targets predicted from ChIP-Seq data, and the y axis gives the frequency that these predictions were verified by ChIP-chip. We plot data for promoters that are predicted by dIP and not MACS, MACS but not dIP, and both dIP and MACS. dIP predicted more target genes and its predictions agree better with ChIP-chip predictions. (D) Common gene target predictions from ChIP-chip and ChIP-Seq as a function of decreasing ChIP-Seq binding scores. (E) Jaccard's similarity coefficient was used to compare predicted ETS1-target promoters using 3 replicate IPs and 4 replicate IgG control assays, comparing the average similarity between predictions across IgG controls using the same IP (replicate IgG) or across IP assays with the same IgG control (replicate IP); error bars are given as S.E.M.

**Figure 3:**
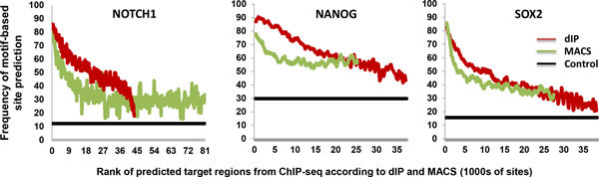
**Comparison of binding site enrichment in predicted target regions**. Frequency of motif-predicted binding sites for NOTCH1, NANOG and SOX2 in dIP and MACS predicted bound regions as a function of dIP and MACS scores; bound regions are identified genome wide and are not restricted to promoter regions.

In general, dIP predicted more target genes than MACS and its predictions were assessed to be both more functionally relevant and reproducible across replicates and experimental technologies (Figures 2, 3). Namely, dIP predicted more NOTCH1, NANOG, SOX2 and FOXA1 targets, and its predicted targets were more likely to be down regulated (NOTCH1, NANOG, and SOX2) or no longer bound (FOXA1) after transcription factor (TF) silencing. A comparison of predicted NOTCH1, NANOG and SOX2 target predictions using ChIP-chip and ChIP-seq suggests that dIP predictions are more likely to be confirmed by ChIP-chip, and a comparison between ETS1 ChIP-seq replicates suggests that dIP prediction are more reproducible.

### ChIP-Seq peaks are enriched in silencing experiments

To check if dIP- and MACS-predicted target genes are indeed functionally regulated, we compared predictions from ChIP-seq [18] with results from differential expression analysis following the RNAi-mediated silencing of NOTCH1, NANOG and SOX2. For these transcription factors, silencing experiments were performed using lentivirus-mediated shRNA in TALL and NT2/D1 cells followed by gene expression profiling. In addition, we compared dIP- and MACS-predicted FOXA1 targets before and after siRNA-mediated silencing of FOXA1 in MCF7 cells [20].

Using a threshold of p-value ≤ 0.05 (Bonferroni corrected) we identified genes that are differentially expressed upon silencing NOTCH1, NANOG and SOX2. The overlap between these differentially expressed genes and target genes identified by dIP was 34%, 26% and 16% of dIP-predicted targets whereas overlap with MACS-identified target genes was 30%, 17% and 13% of MACS-predicted targets, respectively for NOTCH1, NANOG, SOX2 (Figure 2A) suggesting higher sensitivity for dIP-predicted target genes. This suggests that dIP-predicted target genes are more likely to be functionally regulated by the three TFs.

We used MACS and dIP to infer FOXA1 binding sites that are lost following its siRNA-mediated silencing in MCF7 cells. dIP predicted over 2,500 targets in two replicate experiments and fewer than 30% (27% and 21%) of these were retained after FOXA1 silencing, while MACS predicted fewer than 2,000 targets and nearly 40% of them were retained after silencing (39% and 33%); see Figure 2B. These results suggest that dIP has greater sensitivity, as its predicted sites are consistently more dependent on FOXA1 abundance.

### Concordance between ChIP-Seq and ChIP-chip predictions

We studied the concordance between target genes predicated by ChIP-Seq and ChIP-chip by considering how frequently ChIP-Seq-predicted promoters were also predicted by ChIP-chip experiments. For this comparison we used NOTCH1, NANOG, SOX2 target promoters, predicted using × scores [21] from ChIP-chip data with IgG controls in both cases. We partitioned predictions from each method into two: those containing target genes uniquely predicted by each method and those containing common predictions. Target genes identified by dIP but not by MACS (dIP-only) were more abundant and were in better concordance with ChIP-chip predicted target genes than targets identified by MACS but not by dIP (MACS-only), while common predictions outperformed predictions by individual methods for NANOG, but not for SOX2; see Figure 2C. Namely, dIP-only included 2324, 1799 and 670 NOTCH1, NANOG and SOX2 targets, and 30%, 10% and 9% of its predictions were in concordance with ChIP-chip data; MACS-only included 66, 1047 and 184 targets for the three TFs, and only 27%, 6% and 6% of its predictions, respectively, were inferred from ChIP-chip data.

In order to evaluate the relative accuracy of dIP and MACS scoring methods, we ranked predicted target genes for both transcription factors using the highest scoring regions in their promoters. We then evaluated concordance with ChIP-chip predicted target genes by considering the overlap in top *n *predictions from ChIP-Seq, where *n *is varied from 1 to all predictions. Our results suggest that top *n *dIP predicted-targets are in better agreement than top *n *MACS predicted targets for NOTCH1, NANOG and SOX2, when comparing both for the overlap with ChIP-chip; see Figure 2D.

### Reproducibility across multiple replicates

To test the reproducibility of target genes identified by dIP using replicate experiments, we used the ETS1 ChIP-seq experiments [19] containing 3 replicates of IPs and 4 replicates of IgG control assays. Assessment of reproducibility was done using Jaccard's similarity coefficient, which measures the ratio between sizes of the intersection and union of target promoters to compare predictions using the same IP and multiple IgGs (replicate IgG), or the same IgG control for multiple IP assays (replicate IP). Comparing predicted ETS1 target promoters genes on 3 replicate IPs and 4 replicate IgG control assays, suggested that dIP predictions are more consistent (Figure 2E) across multiple replicates compared to MACS predictions; Jaccard's index was consistently at least 10% higher for dIP predicted targets.

### ChIP-Seq peaks are enriched for canonical binding sites

To test if dIP-predicted target regions for NANOG and SOX2 are more likely to contain canonical binding sites, we identified DNA-binding position-specific scoring matrices for NOTCH1, NANOG and SOX2 from TRANFAC [22] and JASPAR [23], as well as *de novo *motifs predicted by DME [24] by using ChIP-Seq data for NANOG and SOX2, whose sites were enriched in peak regions predicted by MACS. Control regions, for each of both transcription factors, were chosen as those where IgG binding was significantly high relative to the given IP according to MACS; these are predicted to be binding-depleted regions.

The TRANSFAC motifs M01111, M01123 and M01125 were most enriched, according to a tenfold cross validation procedure, for MACS-predicted NOTCH1, NANOG and SOX2 binding sites, respectively. To measure enrichment, we optimized the balanced error rate by maximizing the sum of the sensitivity and specificity of site prediction [25]. MACS-predicted regions were enriched for motif sites even at the lowest ranks, and dIP-predicted regions had the highest binding site frequency at top ranking regions. Note that here, regions can be identified anywhere in the genome and are not restricted to promoters.

Our results suggest that dIP-predicted target regions for *all three TFs *are more likely to contain canonical binding sites (Figure 3). Similarly, top scoring regions according to dIP are more likely to contain canonical binding sites than low scoring regions. Similar analysis using the most enriched ETS1 motif (TRANSFAC motif M00743) in the top 1000 MACS-predicted targets for ETS1 dataset suggested comparable ETS1 canonical binding site enrichment for both dIP and MACS target regions (**Figure S1**, Additional file 1).

### PCR validation of ChIP-Seq predictions

**Figure 4:**
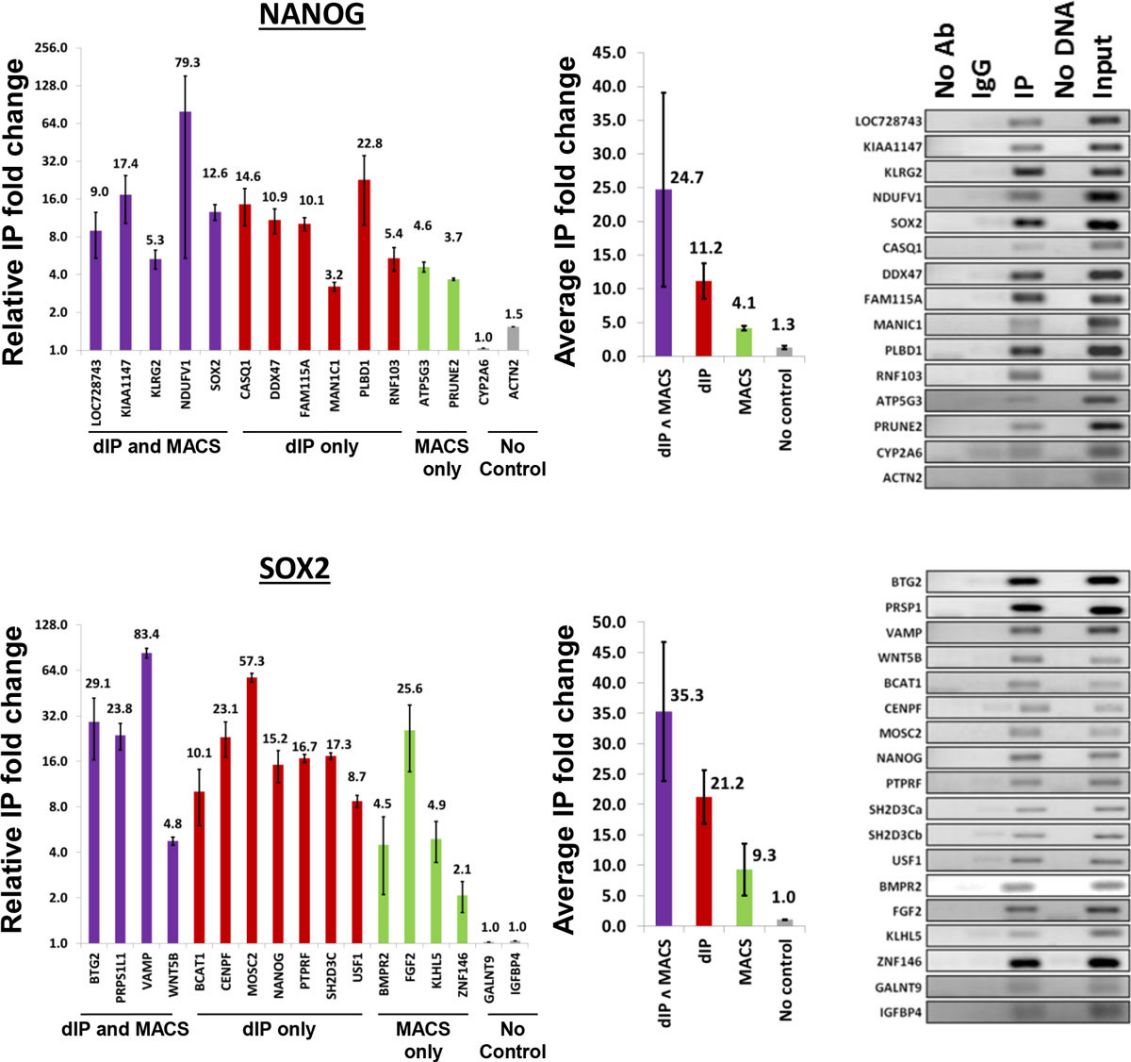
**Verification of promoter binding by PCR**. Top predictions by both dIP and MACS were tested in two biological replicates, including common predictions, dIP-only predictions, and MACS-only predictions. As negative controls (grey) we tested predictions made uniquely by MACS with no control experiment input; error bars are given as S.E.M.

We used ChIP, followed by PCR to test the occupancy of promoter regions that were predicted to bind to NANOG and SOX2 by dIP and MACS; see Figure 4. In total, we tested five and four promoters that were predicted to bind NANOG and SOX2, respectively, by both dIP and MACS; six and seven promoters predicted to bind by dIP but not MACS, two and four promoters to bind by MACS but not dIP. As negative controls, we include results for the top scoring promoters by MACS, two for each of NANOG and SOX2 binding, when controls were not used. In all cases, top predictions in each category were tested. We note that in each of the ChIP-Seq experiments we studied, over one thousand promoters were predicted by MACS to be targeted by each of NANOG and SOX2 only when controls were not used. We expect that the majority of these are false predictions.

The result of our assay shows that dIP-predicted target-regions in promoters have high validation rates in NT2/D1 cells based on individual ChIP-PCR assays of 22 dIP-predicted binding sites of the NANOG and SOX2 transcription factors, including 13 sites predicted by dIP but not by MACS. In addition, dIP detected 9 of 11 and 3 out of 11 targets previously validated for NANOG and SOX2 [18], respectively, while MACS predictions included only 6 of 11 and 2 of 11 of these validated targets, respectively. Taken together, dIP and MACS predicted 12 of 22 (55%) and 8 of 22 (36%) of the validated targets for NANOG and SOX2 reported by Jagadish et. al. [18], respectively.

## Conclusions

We describe a non-parametric method for assigning significance to binding peaks identified from ChIP-Seq data. Its scoring approach is intuitive and fast: a single scan of the data is sufficient to identify genomic regions with potential for binding. Then, these potential binding regions are used to score and annotate full region boundaries.

Our tests suggest that dIP identifies regions, and, consequently, target genes that are missed by MACS, the leading peak caller for ChIP-Seq. dIP-predicted targets are more likely to have evidence for functional regulation as suggested by comparing ChIP-seq data with silencing experiments for NOTCH1, NANOG and SOX2, and are in better agreement with both ChIP-chip and ChIP-PCR (NANOG and SOX2) predictions. Moreover, dIP-inferred TF-bound promoters are more enriched for canonical binding sites of the respective TFs. Furthermore, dIP-predicted ETS1 binding sites are in better concordance with predictions derived from multiple replicate assays. Interestingly, our results suggest that combining dIP and MACS predictions may identify regions with the strongest binding affinity (Figure 2C and 4). In total, dIP predictions showed higher sensitivity, higher specificity, and higher reproducibility, based on replicate experiments, functional assays, and low-throughput validation.

The results of our assays support predictions by both dIP and MACS, but suggest that common dIP- and MACS-predicted promoters are more frequently bound by the two transcription factors tested, and that targets predicted by dIP have stronger binding evidence than targets predicted by MACS and not by dIP. We believe that dIP's main advantage is in the elimination of any reliance on parametric summaries of binding data. Instead, dIP adopts direct control, exclusively relying on experimental data to build its null distributions. In contrast, methods that rely on genome-wide summaries to make predictions are prawn to make both false-positive and false-negative calls in regions with greater and lower non-specific binding, respectively.

ChIP-seq is widely used as an initial screen to identify genes and pathways that are disrupted by changes in expression or activity of transcription factors. For this reason, it is important to reduce both false-positive and false-negative prediction rates. While high false-positive rates will make any technology more difficult to use, high-false negative rates can result in failure to identify key interactions. For example, dIP, but not MACS, predicted MYC targeting by NOTCH1 in TALL cells; a regulatory interaction that is thought to play a key role in T cell acute lymphoblastic leukemia [2,17]. In fact, of 6 validated interactions presented in [2], MACS only predicted 3, compared to dIP's 5. Given the proliferation of ChIP-seq as a screening tool in both industry and academia, we believe that any improvement to its accuracy will help improve our understanding of cellular signaling in all contexts, including development and disease.

## Methods

### dIP algorithm

dIP starts by normalizing the number of IP and control reads. Normalization essentially equates the number of IP and control reads by increasing the size of the smaller set through resampling with replacement. Then it uses a three-step process to identify regions that are enriched for immunoprecipitated fragments and score them. First, dIP evaluates the statistical significance of IP reads in segments of fixed length, based on a comparison between the distributions of mapped reads in experiments using the specific antibody and controls (*e.g*., IgG). Then it merges significant neighboring segments into likely bound genomic regions. In the third and last step, it assigns a score to all the consolidated regions using an intuitive scoring algorithm.

#### Fixed-length segment evaluation

Given an approximate lower bound *L *for the length of immunoprecipitated DNA fragments, dIP scans the whole genome in overlapping window (henceforth called segment) of length L with each successive segment starting at an offset of $+\frac{L}{4}$so that the consecutive segments overlap by 75% (3L/4). In each segment, the number of IP reads (defined as *magnitude, m*) and the number of total reads (defined as *amplitude, a*) is calculated, such that $a=m+\bar{m}$, where ($\bar{m}$) is the number of control reads in that segment. We identify the number of genomic segments, N(a, m), for every ordered pair (a, m). Note that for any particular amplitude a = a_0_, the magnitude m can take any value from the set {0, 1,...a_0_-1, a_0_}, thus generating a distribution of count, N(a0). Next, we calculate the minimum magnitude, *m_min_*, for each amplitude, that is necessary for achieving statistical significance at a given FDR cutoff of φ. *m_min _*is calculated as the minimum value of m for which the following inequality breaks

$$\frac{\sum_{m^{\prime }=0}^{m^{\prime }=m_{min}}N\left(a,m^{\prime }\right)}{\sum_{m^{\prime }=0}^{m^{\prime }=m}N\left(a,m^{\prime }\right)}<\varphi$$

Thus, *m_min _*is the minimum *magnitude *of IP reads required out of the total reads *a *(*amplitude*), for IP reads to be statistically significant in any segment of *amplitude a*. It is obvious that the minimum magnitude is monotonically coupled with the desired FDR cutoff. In addition, we require *m_min _*to increase monotonically with *a*, and this is achieved by setting *m_min _*at amplitude *a*, $m_{min}^{a}=max\left(m_{min}^{a},m_{min}^{a-1}\right)$.

#### Summarization

We choose a gap length *δ*, such that statistically significant segments that are closer than *δ *on the genome can be merged into larger consolidated region predictions. The merged regions thus obtained are checked for overall statistical significance, requiring that the ratio of control reads to total reads in merged region is less than the FDR *φ*. Statistically significant merged regions with minimum length *k *(predefined) are selected as candidate bound regions. Throughout this manuscript, we set *k *= 150 and *δ *= 1000; note that *k *was chosen to match half the expected fragment length (200-300 bases), while *δ *is set wide enough so that a single binding site is not likely to be represented by fragments with this gap length; here two fragments that contain a single binding site are expected to span at most 400-600 bases.

Note that dIP may predict long target regions, with length exceeding 2000 bp; these candidate regions are thought to contain multiple binding sites, and dIP makes no attempt to narrow down their precise location. Instead, given that our focus is on identifying target regions and quantifying the likelihood that these regions are truly bound by the IPed protein, dIP simply rewards these regions by scoring them higher.

#### Scoring

Statistically significant regions are scored as $\sum_{segments}a\;\text{ln}\left(\frac{m}{\bar{m}}\right)$, where $m,\bar{m},a=m+\bar{m}$, are the IP and background counts, and the amplitude, respectively. To get an intuitive understanding of this scoring method, consider the Kullback-Leibler divergence, *D_KL_*(*X*||*Y*), which measures the information loss when Y is used to approximate X. By a little adjustment, the score

$$\sum_{segments}a\;\ln\left(\frac{m}{\bar{m}}\right)=D_{KL}(m||\bar{m})-D_{KL}(\bar{m}||m)$$

In any region, if the IP(m) is highly significant, it will have much more information than control ($\bar{m}$). This is to say that the information loss in approximating the IP as control will be very high, so that the first $D_{KL}\left(m||\bar{m}\right)$ will be high; whereas the second $D_{KL}\left(\bar{m}||m\right)$ will be almost equal to zero as the information content of ($\bar{m}$) is very less, and consequently there will be effectively no information loss when ($\bar{m}$) is coded as m. This will give a high score to the highly significant binding regions. Figure 1A gives *m_min _*for both ChIP-Seq experiments as a function of the amplitude, for *φ *= 0.1. Throughout the work reported here, we set *φ *= 0.1 and *L *= 100.

To account for variability in length between MACS- and dIP-predicted IP and IgG peaks, we set each region length to 800 bases centered at the middle of the peak region; 97% of MACS regions are shorter than 800 bases and so our regions cover MACS-predicted peaks. MACS-1.4.2 was run with default parameters.

### Comparing enrichment for canonical binding

For each chosen motif, and both IP regions and control regions, as predicted by MACS, we used motif class [25] to set optimal score cutoffs. motif class identifies score cutoffs by optimizing balanced-error rates between IP and control region sites, equal to 1-average (sensitivity, specificity). Regions predicted to bind the immunoprecipitated protein were ranked by MACS score and partitioned into 100-region bins. We then recorded the number of regions in each bin that contained at least one predicted site for the motif. The procedure was repeated for dIP-predicted regions, using scores optimized for MACS-predicted targets. As controls, we give the total proportion of control regions that contained sites for the motif.

### Cell culture and immunoprecipitation protocol and analysis

NT2/D1 cell cultures (70-80% confluent) were fixed by adding formaldehyde (37% stock) directly to tissue culture media to a final concentration of 1%. The crosslinking was done for 10' at RT by shaking [18]. The cross-linking reaction was stopped by adding glycine to a final concentration of 0.125 M for 5' at RT with shaking. The cells were washed thrice with ice-cold 1X PBS with 1 mM PMSF (Protease Inhibitor). Cells were scraped from the flask and pelleted by centrifugation. Cross-linked cells were split into 3 × 106 aliquots and were sonicated in TE buffer and Protease inhibitor cocktail (Roche). The sonicated DNA was checked by reversing cross links to get a smear with maximum concentration in 100-200 bp range. The sonicated DNA was immunoprecipitated using anti-NANOG (R&D AF 1997, Minneapolis, MN, USA), anti-SOX2 (R&D AF 2018, Minneapolis, MN, USA) and anti-POU5F1 antibodies (R&D AF1759, Minneapolis, MN, USA) as well as species and isotype-matched control antibodies Goat IgG; R&D, Minneapolis, MN, USA) as control for the specificity, following protocol from Odom et. al. [26]. The precipitated complexes were reverse crosslinked and DNA was purified by phenol-chloroform extraction. The DNA was re-suspended in water for further analysis by PCR. Single end 50-base reads from NANOG and SOX2 ChIP-seq assays and 26-base reads from ETS1 ChIP-seq assays were aligned to hg19 using BWA [27], with duplicates removed before running peak prediction. MACS version 1.4.0 was ran with default parameters.

### ChIP-PCR

PCR primers were designed to sequence regions surrounding the canonical binding sites of the TFs in a 1 Kb promoter region. Oligonucleotide sequences are given for each of the target selected for DIP as well as MACS result comparison below. All the PCRs were performed using 2X Fast SYBR green master mixes (ABI) with 10 ng of precipitated DNA for each sample. The PCR conditions were 95°C-20 sec, 95°C-3 sec and 60°C-32 sec for up to 40 cycles. PCR products were resolved on 2% agarose gels and visualized by ethidium-bromide staining.

#### NANOG

LOC728743, Forward Primer (F) TCTGCTCACCACCTCCGGGA, Reverse Primer (R) TCCATGGGCACAATTGCTCTGCA; KIAA1147 (F) TGAGAACCCCAAAATTACCG, (R) CTGTCAATGTCTCCAGGGCAGCT; KLRG2 (F) CCTGACCCTTCCCTCTTCTT, (R) CCCCAATTCCTTTGGATTCT; NDUFV1 (F) GTCATGAGGATCGGGCTATG, (R) GCCTTTGATGCCACAAGTAAA; *SOX2 *(F) CCCCCAGCAGACTTCACAT, (R) ccctcccatttccCTCGTTT; CASQ1 (F) AGGAGGGAAGGGGCCCTCAGT, (R) TGGGTCAGTTGAGGTGCGGGA; DDX47 (F) TGGTTGTACATGATGGGTCTG, (R) GGCCAAAGGAATAGCTTCAA; FAM115A (F) AGTGTCTGGCACATGGAAAA, (R) GATTTGCAGggaaatgagga; MANIC1 (F) ACATGCCAGTCTCTGTGTGC, (R) GGAGGTGATGTTTCCACTGGCCC; PLBD1 (F) AGTAagaatcctttccAGATTGCTG, (R) CAGTTCCTCCAACAGGAAGC; RNF103 (F) GTCTATCACGCTACATGTCTATAAGG, (R) TCCTGAAAGTGGAATGTAATTTGA; ATP5G3 (F) AAGAAAACggcaatgggtta, (R) CCCTCTCTGGTGCGTAGC; PRUNE2 (F) TAACTCCTGCAGTGGAGCAA, (R) gataaagagcccAGCCTTCTG; CYP2A6 (F) CCAAGGGTGGAGGAGAGAG, (R) CCTCGACATCGTGTTTCTCTG; ACTN2 (F) CACTGGAGAAAGAGGGACAGA, (R) TTCATTGGAGACCCCTCTTC;

#### SOX2

BTG2 (F) GGAGGAAGAGAGGCCAAGTT, (R) CTGCCCAGGACCTCattaga; PRPS1L1 (F) AGCCCTGAATTTGAGAAGCA (R) TTGCTTCAGCTAGCCGAGTT; VAMP1 (F) CattagttatggaaacTGTTCCAAG (R), CCAAGATCTCTctttgggatg; WNT5B (F) GGGCGTGGAAGCTGTTAGT, (R) CCCCCTTTTGATTTTCCTTC; BCAT1 (F) CCCTTTTGTTAGGCCAGCTT, (R)TGATACACATGTAAATGGGATTGGA; BCAT1 (F) CAAAAACGCGTAATTAACCACA, (R) TTTCTGGCTTCCTCATGCTT; CENPF (F) GTTTTCCTCCCCTACCTGCT, (R) TTTTGCATCCCTAGTAACTTTGC; MOSC2 (F) ATGCTGGGCTGCAAATGTA, (R) AGGAACTTTGCCATGGTGAG; *NANOG *(F) AGTCTGGGTTACTCTGCAGCTACt, (R) AACCAGCTCAGTCCAGCAGA; PTPRF (F) AGTTTCCTTTTCCAGCTGCTC, (R) CCAGGGCACACAAAGCTG; SH2D3C (F) GTatttacaaacaaaagAAAGCTGAG, (R) GTTGCTGGGCTGTAGCTTG; SH2D3C (F) CACTCTGTGTTCCCAACCCTA, (R) CTCCTGCAGGCACTGTGTT; USF1 (F) TGCTGCAGAGGAGACAGCTA (R) GTTCCTGACCCCCTGTTGT; BMPR2 (F) TCCCACTCTCTATCCCGACA, (R) GAGAGAGGGCCTGGATTCAC; FGF2 (F) TTGTAACCTGTCCTCCTGTAAGTG, (R) CCCTGTGGGTCTTTTCTCAG; KLHL5 (F) GAACCTACTttttctgattcttatTA, (R) TCTCAGAGCTTAAAGGGAACTCA; ZNF146 (F) CAGTCACACAATCTACTCTCCACTG, (R) GTGACTGGGTGCCGTAAAGT; GALNT9 (F) GTCTGTGTGCCCGTGTGT, (R) CACATGAACAAACAGAGATGAACA; IGFBP4 (F) GCTCTCGAGTTCTGTTTTCCTC, (R) CAGGGAAAGACCAACAGTGA

## List of abbreviations

ChIP: Chromatin Immunoprecipitation; dIP: differential Immunoprecipitation; TF: transcription factor.

## Competing interests

The authors declare that they have no competing interests.

## Authors' contributions

MB, AC and PS conceived the project. MB, SA, and PS designed methods. GM, RK, AI, and RC performed experiments. MB, HRK and MK implemented methods. MB, SA and PS wrote the manuscript.

## Supplementary Material

Additional file 1 **Figure S1. Comparison of binding site enrichment in predicted target regions for ETS1**. Frequency of motif-predicted binding sites for ETS1 in dIP and MACS predicted bound regions as a function of dIP and MACS scores; bound regions are identified genome wide. Click here for file

## Declarations

This supplement has not been supported by sponsorship. A Texas Children's Cancer Center fund (PS) covered publication charges.

This article has been published as part of *BMC Genomics *Volume 16 Supplement 5, 2015: Proceedings of the 10th International Conference of the Brazilian Association for Bioinformatics and Computational Biology (X-Meeting 2014). The full contents of the supplement are available online at http://www.biomedcentral.com/bmcgenomics/supplements/16/S5.
