# Supplementary figures and images for: Direct ChIP-Seq significance analysis improves target prediction
Source: BMC Genomics. 2015 May 26;16(Suppl 5):S4. doi: 10.1186/1471-2164-16-S5-S4 (PMC4460594; doi:10.1186/1471-2164-16-S5-S4)

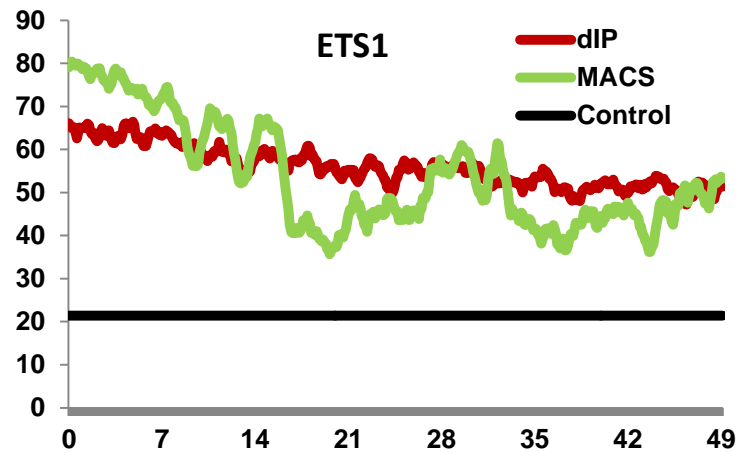

Rank of predicted target regions from ETS1 ChIP-seq according to dIP  
and MACS (1000s of sites)

**Figure S1**

Supplement: Additional file 1 — Figure S1. Comparison of binding site enrichment in predicted target regions for ETS1. Frequency of motif-predicted binding sites for ETS1 in dIP and MACS predicted bound regions as a function of dIP and MACS scores; bound regions are identified genome wide. [file 1471-2164-16-S5-S4-S1.pdf]
